# Soil mycobiome in sustainable agriculture

**DOI:** 10.3389/fmicb.2022.1033824

**Published:** 2022-11-28

**Authors:** Magdalena Frąc, Emilia Silja Hannula, Marta Bełka, Joana Falcao Salles, Malgorzata Jedryczka

**Affiliations:** ^1^Institute of Agrophysics, Polish Academy of Sciences, Lublin, Poland; ^2^Institute of Environmental Sciences, Leiden University, Leiden, Netherlands; ^3^Department of Forest Entomology and Pathology, Faculty of Forestry and Wood Technology, Poznań University of Life Sciences, Poznań, Poland; ^4^Department of Microbial Ecology, Groningen Institute for Evolutionary Life Sciences, University of Groningen, Groningen, Netherlands; ^5^Institute of Plant Genetics, Polish Academy of Sciences, Poznań, Poland

**Keywords:** bioinformatics, dysbiosis, eubiosis, fungal microbiome, microbiome-mediated plant protection, phytopathogens, regenerative agriculture

## Abstract

The soil microbiome contributes to several ecosystem processes. It plays a key role in sustainable agriculture, horticulture and forestry. In contrast to the vast number of studies focusing on soil bacteria, the amount of research concerning soil fungal communities is limited. This is despite the fact that fungi play a crucial role in the cycling of matter and energy on Earth. Fungi constitute a significant part of the pathobiome of plants. Moreover, many of them are indispensable to plant health. This group includes mycorrhizal fungi, superparasites of pathogens, and generalists; they stabilize the soil mycobiome and play a key role in biogeochemical cycles. Several fungal species also contribute to soil bioremediation through their uptake of high amounts of contaminants from the environment. Moreover, fungal mycelia stretch below the ground like blood vessels in the human body, transferring water and nutrients to and from various plants. Recent advances in high-throughput sequencing combined with bioinformatic tools have facilitated detailed studies of the soil mycobiome. This review discusses the beneficial effects of soil mycobiomes and their interactions with other microbes and hosts in both healthy and unhealthy ecosystems. It may be argued that studying the soil mycobiome in such a fashion is an essential step in promoting sustainable and regenerative agriculture.

## Introduction

The use of intensive agricultural production worldwide aims to supply sufficient food for humankind. However, the occurrence of sudden ecological disasters and wars show that this apparently stable situation is actually fragile, but overall, we may generally conclude that food security is growing. These benefits have been achieved at the cost of a damaged natural environment, which is causing increased levels of dissatisfaction with regard to modern industrial agriculture (Frouz and Frouzová, 2022). According to the Food and Agriculture Organization, current food systems account for more than one-third of global greenhouse gas emissions (Crippa et al., 2021; FAO, 2021). The other adverse effect is the high degree of deforestation of areas which are significant to the whole planet, such as tropical rainforests. The recent drastic decrease in the area of ‘the lungs of Earth’ has adversely impacted both our climate and biodiversity (Nobre et al., 2016). Intensive farming with excessive ploughing causes land degradation, including its erosion by water and wind. Moreover, this degradation is also significantly changing the soil structure, water availability, and the availability of numerous macro- and microelements taken from the soil by crop plant monocultures (Beylich et al., 2010). The malfunctioning of current food and industrial systems with the resulting high impact on our climate has led to the novel phenomenon of environmental refugees – people who are displaced due to permanent environmental disasters (Myers, 2002).

The lack of nutrient cycling in intensive, high-input agriculture, and the use of considerable amounts of pesticides, has an impact on the natural soil microbiome, including its mycobiome (O’Brien, 2013). The soil mycobiome, which is also called the fungal microbiome, is one of the main components of an integrative microbiome which includes different microbial groups which inhabit agroecosystems (Geisen, 2021). At present, the soil mycobiome is still being poorly studied in relative terms as the main focus of recent global research has been connected to the bacterial component of the soil microbiome (Yadav et al., 2021). Despite the limited research undertaken to date, it is well known that fungi support many ecosystem processes and perform functions that are essential for the sustainable development of future agriculture (Fernandes et al., 2022), including plant–soil interactions, organic matter decomposition (Vétrovský et al., 2019), plant health promotion and nutrition (Põlme et al., 2020). A great majority of the agricultural land currently in use requires soil regeneration and the restoration of its biological processes (Gosnell, 2022), which includes restoring all components of the soil mycobiome. The solution to soil restoration in an agricultural setting is environmentally-friendly farming, which is usually referred to as sustainable agriculture. A holistic approach linking agricultural production with an unharmed environment is referred to as regenerative agriculture (Newton et al., 2020).

A clean environment and the maintenance of economic profitability are significant concerns in agriculture, horticulture and forestry (Dubey et al., 2019). Plant pathogenic fungi decrease plant yield and soil quality (Malarczyk et al., 2019), but beneficial fungi have the potential to contribute to soil stability and functioning and thus serve as vital components of regenerative agriculture. Below we provide an overview of mycobiome-based solutions and tools for sustainable and regenerative agriculture. The thrilling perspective is the possibility of neutralising plant diseases by controlling and altering mycobiome shifts in soils. The microbiome-mediated plant protection is an emerging direction of research (Bass et al., 2019), shifting the traditional ‘one pathogen – one disease paradigm’ towards pathobiome healing with microbial inoculants (Berg et al., 2021). Recent studies show that microbial communities in soils are much more diverse than we have previously thought. If so, is the control of myriads of soil microorganisms possible to any extent?

### Soil mycobiome – from butterfly effect to Ariadne’s thread

Microbes play essential roles in the ecology and physiology of plants (Dastogeer et al., 2020). Plants and their associated mycobiomes, including soil mycobiota, form complex and dynamic mutual interactions where the plants provide ecological niches and easily utilizable carbon to the mycobiome, which in turn influence plant growth, development and fitness (Vincent et al., 2020). The rhizosphere mycobiomes and plant endophytes suppress disease (Mendes et al., 2011; Frąc et al., 2018) and improve abiotic stress resistance (Lau and Lennon, 2012; Coleman-Derr and Tringe, 2014).

Fungi in the soil and rhizosphere operate in interlocking networks (van der Heijden and Hartmann, 2016), and form mycological hubs in the soil or in association with plants. The application of practices that promote the complexity and connectivity of these networks may serve to enhance the services that the mycobiome provides and this also has the potential to improve agricultural productivity due to positive soil feedback. Therefore, sustainability involves a greater degree of plant reliance on the beneficial functions offered by the soil mycobiome. In sustainable and regenerative agriculture, the dynamic interactions between soil mycobiome, plant mycobiome and plant health should be linked to agricultural practices (EASAC, 2022).

**Figure 1:**
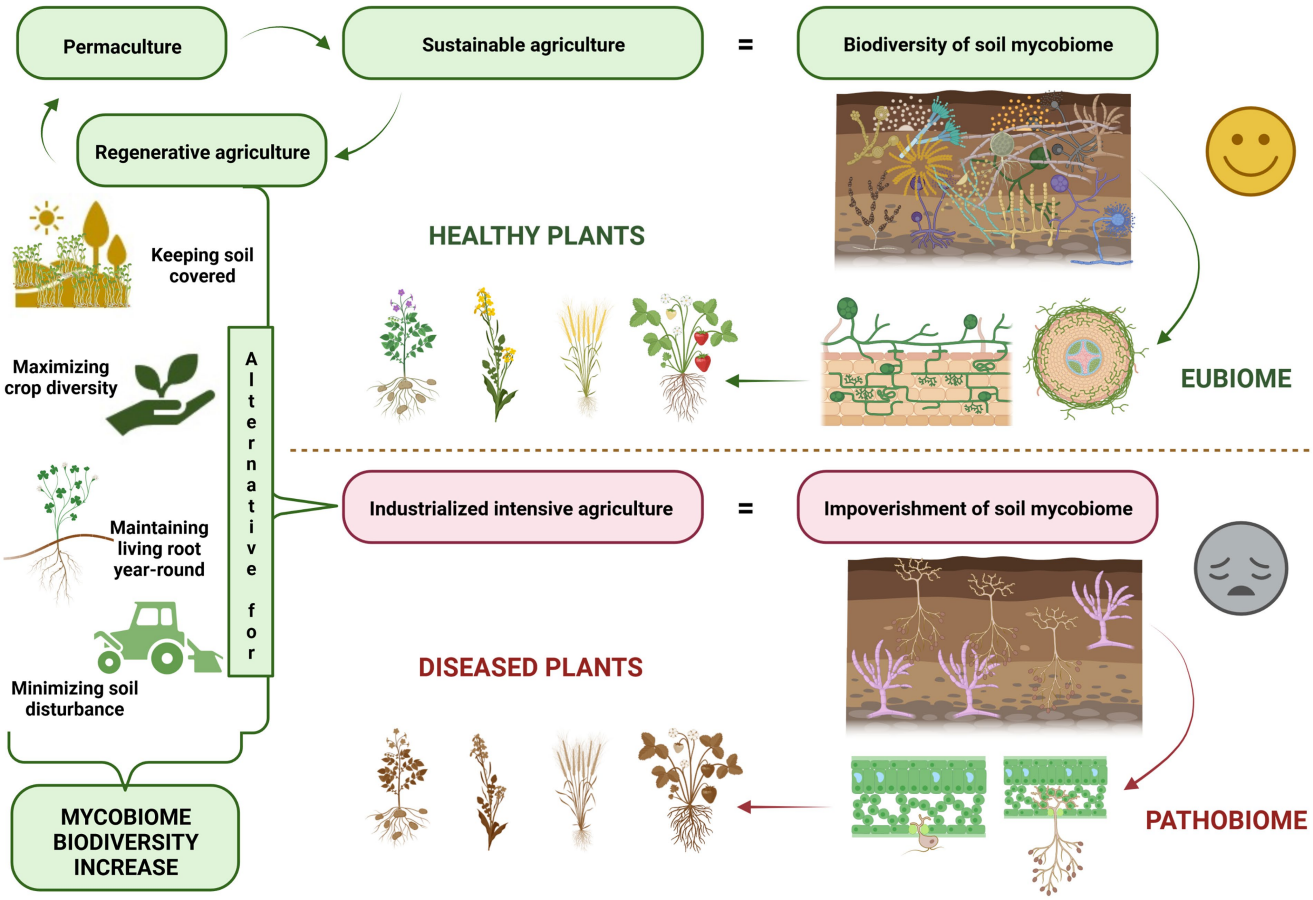
Soil mycobiome plays a vital role in sustainable agriculture, horticulture, and forestry.

Microbial ecosystem balance, which is referred to as eubiosis, is accompanied by a high degree of suitable microorganisms (Iebba et al., 2016) and it may be regarded as a fundamental concept of ecosystem stability (Figure 1). According to this concept pathogens represent a small but important part of the microorganisms present. Their presence may lead to the loss of biodiversity and the dysbiosis state, which shifts microbe composition and influences pathogen emergence and outbreaks (Chen et al., 2020). Healthy balanced host–microbe interactions of the holobiont are termed eubiosis. At the same time, dysbiosis (pathobiome) refers to a holobiont disease state (Berg et al., 2020). Dysbiosis refers to the reduced capacity of host-microbiome regulation. This loss of function causes a decline in plant health (Arnault et al., 2022).

Dysbiosis can be observed not only when plant disease is advanced but even at the initial stage, before planting, and it can predetermine future plant health (Wei et al., 2019). Detrimental mycobiota identified on time may be neutralised using various tools, leading to microbiome-mediated plant protection. Pathobiome analyses showed changes in community structure caused by pathogens (Dastogeer et al., 2022; Jia et al., 2022), and these small differences trigger great micro- and mycobiome modulations (Berg et al., 2021). Van Dijk et al. (2022) proved that soil microbiomes change aboveground plant-pathogen-insect interactions. In other words, small changes can have a great, non-linear impact on a complex soil microbiome-plant health system, referred to as the ‘butterfly effect’.

Because fungi are important organisms around the world (O'Hanlon, 2017), this concept may be used in the context of mycobiomes, thereby indicating that soil and plant mycobiota determine both the healthy and unhealthy states of agroecosystems. The best example of this is suppressive and conducive soils (Schlatter et al., 2017), in which plants actively promote the selection of beneficial microorganisms which are active against soilborne pathogens (Weller et al., 2002). The application of soil amendments to supply the missing parts of the microbiota, such as Streptomycetes, has received considerable attention (Wiggins and Kinket, 2005a,b; Li et al., 2020). Recent studies have shown that the application of biofertilizers actively induces soil suppressiveness, thereby promoting the control of both bacterial and fungal diseases in tomatoes (Deng et al., 2021, 2022). Modulation of the microbiome using microbial inoculants is a promising tool for smart sustainable agriculture (Berg et al., 2021). Soil mycobiome networks undergo complicated dependencies and a great role of science is to disentangle them and use fungal mycelium as Ariadne’s thread into the desired direction, to obtain a healthy crop.

### Beneficial fungi for agricultural crops, fruits, and forests

Among the pathogens of crops, fungi are the most numerous and damaging (Agrios, 2005). A decrease in yield caused by fungi has been documented for all globally important crops, such as wheat, rice, maize, potato, soybean, rapeseed and many others (Savary et al., 2019), this state of affairs has attracted a great deal of attention and caused concern (Jeger et al., 2021). Each crop has at least a few pathogens; while some have several of them, some are important on a worldwide scale and others are damaging at a regional or local scale; this is usually linked to conducive weather conditions. Most of them inhabit the soil and form their mycobiota for at least a part of their life cycle. Moreover, novel pathogens may emerge (Havis et al., 2015) because of adverse climate change, agricultural zones, crop diversity and changes in agronomic practices. Due to the Green Deal policy of the European Union (European Commission, 2020) and the general trend in favour of sustainable agriculture, the current focus of research, and development concerns beneficial microorganisms or their communities, including fungi. At present, the list of beneficial fungi in widespread use in agriculture is short and usually limited to Biological Control Agents developed for horticultural production, typically the generalists which are common in many habitats (Gostinčar et al., 2019) with mycoparasitic properties (Kubicek et al., 2011). The most popular preparations are based on *Trichoderma harzianum* (Błaszczyk et al., 2014; Dawidziuk et al., 2016), *Clonostachys rosea* (Peng et al., 2011; Sun et al., 2020), *Aureobasidium pullulans* (Prasongsuk et al., 2018) and other yeast-like fungi. A good example is *Coniothyrium minitans*, the super parasite of *S. sclerotiorum* (Smolinska and Kowalska, 2018).

Soft fruits are affected by various diseases caused by fungal pathogens inducing damage to roots, leaves, crowns and fruit. The most common and damaging diseases of soft fruit include grey mould, anthracnose, crown and root rot, plant wilt and fungi causing postharvest losses (Gubler and Converse, 1993; Garrido et al., 2016; Malarczyk et al., 2020). In recent years, under the pressure of a changing climate, pathogens which in the past, typically occurred mainly in tropical and Mediterranean climate zones, began to appear in the continental climate zone (Debode et al., 2011; Karimi et al., 2016; Torbati et al., 2019; Malarczyk et al., 2020). Key groups of beneficial mycobiota in soft fruit production include arbuscular mycorrhizal fungi (AMF), which for the most part, belong to the orders Glomerales, Archaeosporales, Paraglomerales and Diversisporales (Boyer et al., 2016; Begum et al., 2019). Ericoid mycorrhizal fungi can improve plant resistance to diseases (Jansa and Vosátka, 2000; Bizabani and Dames, 2015; Fehrer et al., 2019; Ważny et al., 2022). Many biopesticides are based on the entomopathogenic fungi (Bamisile et al., 2018; Canassa et al., 2020; Litwin et al., 2020). Fungal endophytes, such as *Clonostachys rosea* (Cota et al., 2009), *Piriformospora indica* (Sinclair et al., 2013), *Xylaria* sp. (Ważny et al., 2022), *Penicillium* sp. (Hamim et al., 2017), *Hannaella coprosmae* and *Oberwinklerozyma straminea* (Nguyen et al., 2021) are used in biocontrol and can have a beneficial impact on fruit yield (Murphy et al., 2019). The most common fungi able to promote plant growth and capable of acting as biocontrol agents belong to *Trichoderma* (Chen et al., 2019; Pylak et al., 2019; Lombardi et al., 2020; Mącik et al., 2020; Oszust et al., 2021; Rees et al., 2022) and yeasts (Sun et al., 2018; Calderon et al., 2019; De Miccolis Angelini et al., 2019; Freimoser et al., 2019).

Fungi constitute a significant fraction of the forest soil microbiome (Pérez-Izquierdo et al., 2021). Their high degree of ecological and taxonomical diversity significantly impacts the functioning of forest ecosystems (Witzell and Martín, 2018). In forests, saprotrophs, endophytes, biomass degraders, and the decomposers of lignified plant materials perform multiple tasks and form mutualistic relationships with roots (Frąc et al., 2018; Yan et al., 2018; Li et al., 2021). They can positively influence tree health by acting as growth-promoting or protective agents. However, many fungal species are causal agents of forest diseases, although only a few are active pathogens (Witzell and Martín, 2018). Nevertheless, the boundaries between the categories of fungus according to their function are not strictly defined, as some species can shift from one trophic state to another (Delaye et al., 2013).

Fungi are not homogeneously distributed in the forest soil (Lladó et al., 2018). Their activity is driven by the dynamics of ecosystem processes, root exudates, and soil heterogeneity (Baldrian, 2017; Lladó et al., 2018). Communities of litter saprotrophic and root-associated fungi are vertically separated within profiles. Within these layers, the fungal functional groups occupy different spatial niches according to their mode of C assimilation. Also, the competition for space and resources is an essential determinant of these communities (Boddy, 2000; Kennedy, 2010; Bödeker et al., 2016). Changes in climatic conditions, atmospheric CO_2_ levels, forest management regimes, and nutrient availability may affect the various fungal functional guilds in different ways, leading to shifts in their competitive balance and niches (Bödeker et al., 2016). Decomposers dominate the litter layer, while mycorrhizal fungi dominate the humus and mineral layers (Asplund et al., 2019). Few studies have documented the microbial changes that occur after the emergence of a plant pathogen, especially in forest ecosystems (Stewart et al., 2021). Some species (e.g., *Laccaria laccata*, *Hebeloma crustuliniforme*, *Paxillus involutus*, *Trichoderma harzianum*, *Pisolithus tinctorius*, *Tricholoma pessundatum*) have been shown to play an essential role in decreasing the severity of disease caused by root pathogens (Chakravarty and Hwang, 1991; Suárez et al., 2018; Stewart et al., 2021; Zaki et al., 2021). However, some fungal pathogens can deactivate genes in non-pathogenic species; they are involved in antimicrobial production and further weaken tree symbionts by releasing secondary metabolites (Stewart et al., 2021).

### Bioinformatics for mycobiome control in sustainable agriculture

**Figure 2:**
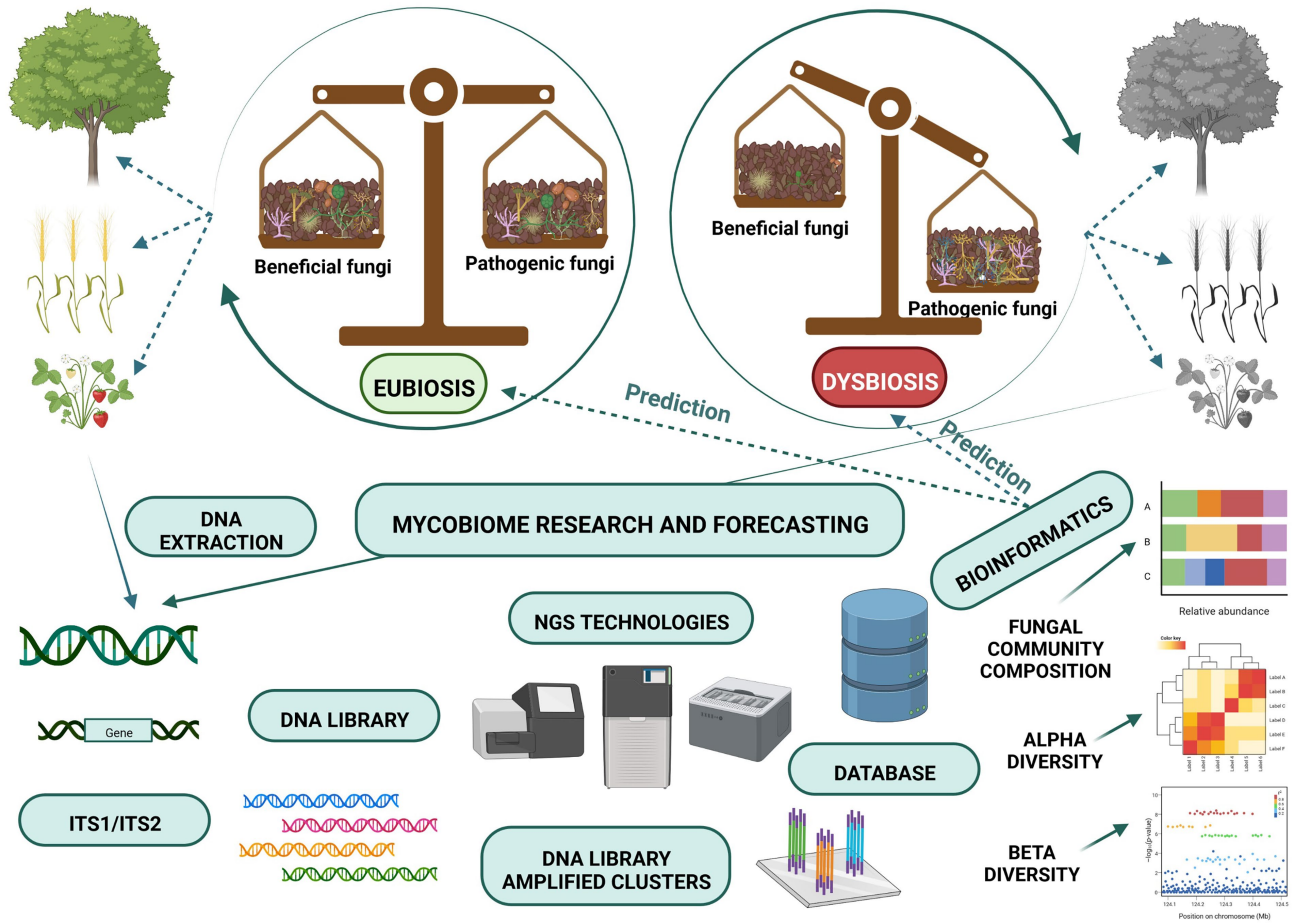
Next Generation Sequencing and bioinformatic programs are modern tools for the monitoring of soil mycobiome composition.

In recent years the development of high-throughput sequencing has enabled scientists around the globe to identify soil fungi (Tedersoo et al., 2014); see Figure 2. Most of the published studies use Illumina MiSeq to characterize the fungi targeting ITS regions, particularly ITS2 (Schoch et al., 2012), which is also deemed to be the best technique in comparative studies (Tedersoo et al., 2015; Frau et al., 2019). There has, however, been a recent call to use longer-read sequencing such as PacBio or Nanopore sequencing and to analyse both ITS2 regions and parts of the 18S and 28S genes (D’Andreano et al., 2021; Runnel et al., 2022). Obtaining longer-read will enable scientists to assign reads to particular species and strains with an increased level of confidence. This will be crucial, especially when linking the identity of a species to its functionality. There are multiple options for bioinformatic analyses and pipelines, and the appropriate choice depends mainly on the sequencing technique used but also on the individual preferences of the scientist. The most commonly used pipelines for the analyses of bacteria and fungi are DADA2 (Callahan et al., 2016) and QIIME2 (Bolyen et al., 2019), which both rely on user-making decisions concerning quality filtering and error learning which is based on the region amplified and organism studied.

Some pipelines have been designed explicitly for fungi (such as PIPITS; Gweon et al., 2015) in which some decisions and steps are automated. For fungi, it is always advised to extract the ITS region amplified after joining pair-end reads to increase the quality of the data (Bengtsson-Palme et al., 2013). As the ITS region is variable in length (Schoch et al., 2014), the step of filtering reads of a specific size should be omitted. Using a curated database like UNITE (Nilsson et al., 2019) is highly recommended when studying fungi. UNITE relies on the species hypothesis concept, which clusters similar sequences into one hypothetical taxonomic unit. After the sequencing data is paired, filtered, clustered, and assigned the appropriate taxonomy, the hypothesis can be tested using multivariate statistical analyses.

Similarly to bacteria, transformations of data and careful interpretation are required to reach reliable conclusions (Weiss et al., 2017; Knight et al., 2018). Unique to fungi, potential functional guilds (such as pathogens, mutualists and saprotrophs) may be assigned using Funguild (Nguyen et al., 2016) or FungalTraits (Põlme et al., 2020) and the richness and relative abundance of these groups can then be evaluated (Hannula and Träger, 2020). However, for many sequence types or taxonomic units, no information concerning their lifestyle is available, thereby rendering this prediction uncertain. Future studies should focus on the functioning of fungal species as well as the functional sequencing of the soils to shed light on the functional diversity of fungi in the soils.

## Concluding remarks

Mycobiomes comprise a large part of the Earth’s biodiversity and play a key role in soils, where they perform numerous functions within the ecosystem. Soil fungi play a crucial role in the environment, and affect plant health as symbiotes, pathogens, or through matter degradation. Agroecosystem mycobiomes are increasingly recognized as beneficial to soil and plant health as they facilitate and even control numerous ecosystem processes. In order to meet the various challenges of maintaining food security and the environment, mycobiome studies connected with plant pathology and protection should implement multidisciplinary approaches, including the use of traditional, molecular and bioinformatic tools. Further research into the identity, abundance, distribution and function of soil mycrobiomes, as well as their different roles in soils, is necessary to understand the dimensions of fungal biodiversity, its impact on plant health and to prevent fungal diseases. It is essential to focus on mycobiome shifts caused by climate change, their interactions with other microbes, and the determining relations between mycobiomes and microbiomes in both healthy and dysfunctional conditions.

The challenge for the future is not only to gain the ability to rapidly recognize the major and trace components of the soil mycobiomes and their functions within networks, as well as their interplay with plant roots and other constituents of the soil but also to gain the ability to redirect them into the desired composition and proportions, thereby healing the ecosystem in question. The coming years will show whether it is just a dream or the first solutions of this kind will start functioning in practice.

## Author contributions

MF, EH, JS, MB, and MJ wrote, drafted, read, corrected, improved, revised and accepted the last version of manuscript. All authors contributed to the article and approved the submitted version.

## Funding

This work was supported by the following projects: BIOSTRATEG3/344433/16/NCBR/2018 funding by The National Centre for Research and Development within the framework of the BIOSTRATEG Programme, SUSCROP/I/POTATOMETABIOME/01/2019 funding by The National Centre for Research and Development within the framework of the ERA-NET SusCrop Programme and the paper was partially financed within the framework of Ministry of Science and Higher Education programme ‘Regional Initiative of Excellence’ in years 2019-2022, Project No. 005/ RID/2018/19’.

## Conflict of interest

The authors declare that the research was conducted in the absence of any commercial or financial relationships that could be construed as a potential conflict of interest.

## Publisher’s note

All claims expressed in this article are solely those of the authors and do not necessarily represent those of their affiliated organizations, or those of the publisher, the editors and the reviewers. Any product that may be evaluated in this article, or claim that may be made by its manufacturer, is not guaranteed or endorsed by the publisher.
